# Species Distribution Models of Tropical Deep-Sea Snappers

**DOI:** 10.1371/journal.pone.0127395

**Published:** 2015-06-01

**Authors:** Céline Gomez, Ashley J. Williams, Simon J. Nicol, Camille Mellin, Kim L. Loeun, Corey J. A. Bradshaw

**Affiliations:** 1 Oceanic Fisheries Programme, Secretariat of the Pacific Community, BP D5, 98848, Noumea, New Caledonia; 2 The Environment Institute and School of Biological Sciences, The University of Adelaide, Adelaide, South Australia, 5005, Australia; 3 Australian Institute of Marine Science, Townsville, Queensland, Australia; Aristotle University of Thessaloniki, GREECE

## Abstract

Deep-sea fisheries provide an important source of protein to Pacific Island countries and territories that are highly dependent on fish for food security. However, spatial management of these deep-sea habitats is hindered by insufficient data. We developed species distribution models using spatially limited presence data for the main harvested species in the Western Central Pacific Ocean. We used bathymetric and water temperature data to develop presence-only species distribution models for the commercially exploited deep-sea snappers *Etelis* Cuvier 1828, *Pristipomoides* Valenciennes 1830, and *Aphareus* Cuvier 1830. We evaluated the performance of four different algorithms (CTA, GLM, MARS, and MAXENT) within the BIOMOD framework to obtain an ensemble of predicted distributions. We projected these predictions across the Western Central Pacific Ocean to produce maps of potential deep-sea snapper distributions in 32 countries and territories. Depth was consistently the best predictor of presence for all species groups across all models. Bathymetric slope was consistently the poorest predictor. Temperature at depth was a good predictor of presence for GLM only. Model precision was highest for MAXENT and CTA. There were strong regional patterns in predicted distribution of suitable habitat, with the largest areas of suitable habitat (> 35% of the Exclusive Economic Zone) predicted in seven South Pacific countries and territories (Fiji, Matthew & Hunter, Nauru, New Caledonia, Tonga, Vanuatu and Wallis & Futuna). Predicted habitat also varied among species, with the proportion of predicted habitat highest for *Aphareus* and lowest for *Etelis*. Despite data paucity, the relationship between deep-sea snapper presence and their environments was sufficiently strong to predict their distribution across a large area of the Pacific Ocean. Our results therefore provide a strong baseline for designing monitoring programs that balance resource exploitation and conservation planning, and for predicting future distributions of deep-sea snappers.

## Introduction

Understanding spatial and temporal patterns of species’ distributions is fundamental for assessing threats to biodiversity and developing appropriate conservation management measures [1]. Protected areas have become a prominent conservation tool for marine ecosystems, with decisions about their location, size and number informed by the distribution of species and habitats of perceived ecological importance, vulnerability or rarity [2].Such spatial planning is also a feature of the *Convention on Biological Diversity*, including the identification of ecologically or biologically important marine areas in need of protection in open-ocean waters and deep-sea habitats [3].

Marine protected areas are most common in coastal and shallow water habitats, where they have had demonstrable benefits for fisheries and biodiversity conservation worldwide [4–6]. However, the shift in global fishing pressure towards deeper waters since the 1950s [7, 8] highlights the need for complementary efforts in spatial management of these habitats [9]. The impending challenge will be the collection of sufficient data from deep-sea habitats, because spatial information for resident species is patchy or non-existent due to the vast area, remoteness, and expense of exploration relative to shallow, coastal waters. Species distribution modelling provides an opportunity to extrapolate from existing spatially limited data to the entire potential distributional range of species [10–12], and could be particularly useful for predicting species’ distributions in deep-sea environments [13].

Deep-sea fisheries occur within all ocean basins and typically concentrate on seamounts, continental slopes and other deep bathymetric features. Life histories of most deep-sea fishes are characterized by high longevity, slow growth, delayed maturity, and low fecundity, indicating low production potential and resilience [8, 14–16]. As such, many deep-sea species are considered more vulnerable to exploitation than shallow-water species [8, 14, 17], placing greater urgency for conservation planning in the deep-sea environment.

In the tropical and sub-tropical regions of the Pacific Ocean, most deep-sea fisheries are small-scale artisanal and subsistence fisheries that have strong local economic and cultural value in many Pacific Island countries [18, 19]. These small-scale fisheries operate along deep reef slopes and near shallow seamounts and banks at depths ranging approximately 100–400 m. While this depth range is shallower than that typically applied to the suite of long-lived, deep-sea species (400–2000 m), it is considerably deeper than the adjacent shallow water coral reef and lagoon fisheries (0–50 m) characteristic of Pacific Island countries. Furthermore, small-scale deep-sea fisheries target species of snapper (Lutjanidae), grouper (Epinephelidae) and emperor (Lethrinidae) that occupy the greatest depths within each family, and that have similar life history characteristics to other long-lived, deeper-water species [20, 21]. In this context, we consider these small-scale fisheries to be ‘deep-sea’. Given the potential vulnerability to exploitation of these deep-sea species, based on their life history traits [22], developing management strategies to ensure ecological and economic sustainability of such fisheries has become a priority for Pacific Island resource managers [19].

Rapid expansion in the number of vessels operating in these fisheries occurred during the 1970s, but was soon followed by declines in fishing effort only two decades later, mainly due to declining catch rates, unreliable access to export markets, and a shift towards tuna long-lining, which was more profitable at the time [18]. More recently, there has been interest in re-developing deep-sea fisheries in the Pacific in recognition of the limited potential for further commercial exploitation of shallow reef and lagoon fisheries in the region [22], and stakeholders’ perceptions that unexploited populations exist in more distant locations. However, policy makers are approaching such opportunities with caution because there are currently few countries with management plans that encompass deep-sea fisheries, and there are few data available to support policies for sustainable exploitation or conservation.

Across the Western Central Pacific Ocean, at least six countries have active deep-sea snapper fisheries or have participated in such fisheries historically, and at least fourteen countries have expressed some interest in developing this capacity [18].It is plausible that many of these nations are exploiting the same stocks, given the wide distribution of most target species, and lack of genetic structure in eteline snappers at large spatial scales [23]. Consequently, collaboration among countries [24] based on a consensual distribution map of deep-sea snapper habitats [25] could provide the basis for better spatial management of these target species in the region. To this end, we constructed species distribution models for deep-sea snappers using spatially limited presence data for the main harvested species in the Western Central Pacific Ocean. We identified the physical and oceanographic variables most influential in determining distributions of these species at broad spatial scales (1000s km), and used an ensemble modelling approach to predict the distribution of these species across the region.

## Methods

### Study area

Our study area was the Western Central Pacific Ocean between 15°N and 30°S and 120°E and -170°W, representing almost 20 million km^2^, and encompassing the Exclusive Economic Zones of 32 countries and territories. The reference bathymetry layer 1-minute Gridded Global Relief Data, ETOPO1, [26] was validated across the study area through compilation with local, accurate and high-resolution bathymetry layers in New Caledonia and Tonga, and with the global distribution of seamounts representing approximately 4.7% of the ocean floor [27].

### Study species

Deep-sea snappers (sub-Family Etelinae) are comprised of five genera (*Aphareus*, *Aprion*, *Etelis*, *Pristipomoides* and *Randallichthys*)and at least 19 species [28]. *Aprion* and *Randallichthys* are monotypic genera, while there are two species of *Aphareus*. *Etelis* and *Pristipomoides* are the most speciose (4 and 11 species, respectively), and comprise the most commonly exploited species. The most common species captured in the Western Central Pacific Ocean deep-sea fisheries are *Etelis carbunculus*, *E*. *coruscans* and *E*. *radiosus*, which are usually captured between 200 and 400 m deep, and *Pristipomoides filamentosus*, *P*. *flavipinnis*, *P*. *multidens*, *P*. *zonatus*, *P*. *seiboldii*, *P*. *argyrogrammicus* and *P*. *auricilla*, which are typically captured in shallower water between 50 and 300 m [29]. Of the other deep-sea snapper species, only *Aphareus rutilans*(commonly captured between 50 and 400m) are frequently captured and reported.

### Deep-sea snapper distribution data

We collated presence-only data for deep-sea snappers from several sources including (*i*) research surveys across the territorial waters of 19 countries of the Western Central Pacific Ocean done by the Secretariat of the Pacific Community from 1979 to 1988 [18], and in 2012 (A. J. Williams, Secretariat of the Pacific Community, New Caledonia, unpublished data), (*ii*) research surveys and commercial fisheries data collected in New Caledonia from 1979 to 1995 [30], (*iii*) commercial fisheries logbook data reported to the New Caledonian Provincial Governments from 2000 to 2008, and (*iv*) commercial fisheries logbook data reported to the Tongan Government from 2005 to March 2012. Fisheries data from other countries were either unavailable or unreliable. While some datasets distinguished individual species, many did not. Therefore, we grouped species within each genus (*Etelis*, *Pristipomoides* and *Aphareus*) for all data to provide a consistent dataset, recognizing the similarity in depth preference among species within each genus [31]. As a consequence, variation in biological or ecological characteristics among species within each genus could not be captured by our models, and thus predictions represent the potential distribution at the genus level.

### Physical and oceanographic data

We derived explanatory variables from physical and oceanographic data based on their biological relevance, resolution and availability. We considered that the distribution of deep-sea snappers would be most influenced by depth, bathymetric slope and temperature, based on analyses of underwater video camera data for the same species from the main Hawaiian Islands [31, 32]. Physical data were represented by bathymetry from which we extracted the depth (m) and slope (%) from global bathymetry (ETOPO1) at 0.016° [26]. More precise bathymetry data were available for New Caledonia at 0.00045° resolution [33] and Tonga at 0.0045° resolution [34]. We merged the bathymetry datasets at a consistent resolution of 0.016°, conserving the values of the higher-resolution datasets where available.

Temperature at depth was available from two datasets with different spatial extents and resolutions: (*i*) Regional Oceanic Modeling System—*ROMS*—[35], available from 10 to 30°S and 145 to 190°E at 0.083° resolution and (*ii*) Pacific-wide climatological data available at 0.25° resolution [36]. We used the ROMS data in preliminary models covering a smaller geographical area, while we used the climatological dataset in broader-scale regional distribution models (see below). Monthly averaged temperature (1999–2006) from the ROMS data was available at 36 specific depth layers from 2 to 4589m. The climatological dataset provided averaged monthly temperature (2001 to 2012) at 46 specific depth layers from 3 to 5875m.Temperature data at depths > 100 m were often not available at the boundaries of land masses and shallow reefs because of insufficient spatial resolution. Because much of the species’ presence data were within these boundaries, we used only temperature data from 3–100 m. Spatial correlations (data not shown) revealed that the spatial patterns in temperature at these depths were indicative of spatial patterns at greater depths that deep-sea snappers also inhabit. We aggregated the depth layers from each dataset over two depth intervals (3–50 m and 50–100m) by computing corresponding depth-weighted means, attributing the width of each depth layer as the weight.

### Distribution modelling

We constructed species distribution models to predict the distribution of deep-sea snappers in the Western Central Pacific Ocean. We grouped presence-only data (i.e., ≥1 individuals) into three species groups: *Pristipomoides*, *Etelis*, and *Aphareus*. First, we developed preliminary models calibrated independently on datasets from two different locations to evaluate the influence of quality and precision of input data on predictions. We chose New Caledonia and Tonga for the preliminary modelling because fisheries-dependent data, for which locations are likely to be more imprecise than research survey data, were available only from these locations. Second, we implemented regional distribution models using all available presence data to predict distributions of each species group across the Western Central Pacific Ocean.

For all models, we used four different algorithms available within the BIOMOD framework [37] implemented in the R package, to obtain an ensemble of predicted distributions: classification tree analysis (CTA), generalized linear models (GLM), multiple adaptive regression splines (MARS) and MAXENT (V3.3.3k). We chose this restricted set of algorithms because they perform well across a range of scales and situations [1, 37, 38] and it was beyond our scope to evaluate the complete range of available algorithms. BIOMOD provides a useful interface to compare and assemble presence-only species distribution modelling methods to predict species distributions [39].

To calibrate the models and to evaluate their performance, we did a five-fold cross-validation (implementing five replicated runs) by partitioning the presence dataset into 70% to calibrate and train the models, and 30% to validate and test the predictions. Validation data were selected randomly for each independent model leading to a random and extended geographic coverage. We evaluated each independent model according to the true skill statistics (*sensitivity + specificity -1*), which is a simple and intuitive measure for the performance of predictive maps [40]. We estimated variable contributions during independent modelling by re-sampling each explanatory variable 100 times during the modelling process. We used ensemble modelling to combine independent model outputs, evenly weighted, with at least a true skill statistic of 0.7 (keeping the most accurate models) and to provide an ensemble prediction [37]. We generated global ensemble models by combining independent models based on all pseudo-absence datasets, all algorithms and all repetitions. We implemented four ensemble-model metrics: mean of probabilities, coefficient of variation of probabilities (corresponding to the standard-deviation/mean ratio), median of probabilities and weighted mean of probabilities. We evaluated ensemble predictions using true skill statistics considering all pseudo-absences as absences.

### Preliminary models

**Fig 1:**
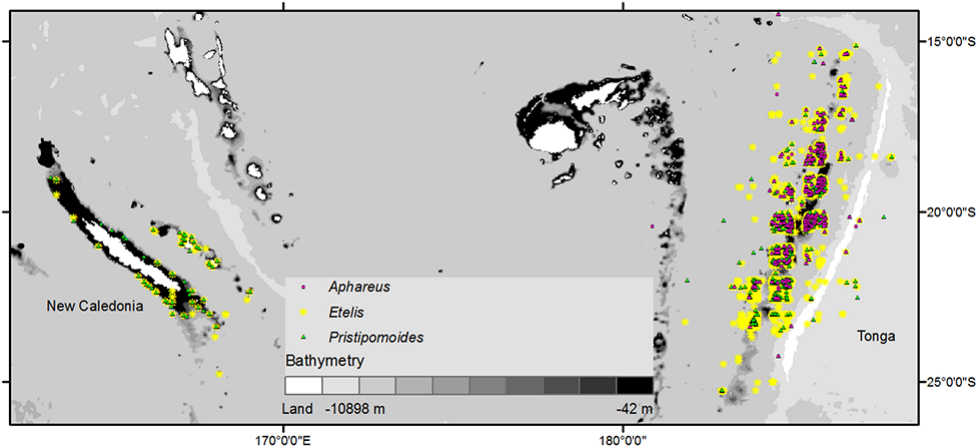
Location of presence data for deep-sea snapper in New Caledonia and Tonga.

We compared predictions from preliminary models calibrated independently on datasets from New Caledonia and Tonga (including both fisheries-dependent and research survey data). We used presence data for *Etelis* and *Pristipomoides* only because *Aphareus* was not recorded separately in New Caledonia. In New Caledonia, presence data were available from 52 locations for *Etelis* and 56 locations for *Pristipomoides* (Fig 1). In Tonga, presence data were available from 1465 locations for *Etelis* and 817 locations for *Pristipomoides* (Fig 1).

We used ensemble modelling to predict the distribution of each species group in New Caledonia (or Tonga) based on the predictive preliminary models calibrated across Tonga (or New Caledonia). We generated three sets of pseudo-absences with 50% more records than presence occurrences and geographically restricted around presence records to delineate more precisely the margins of suitable versus unsuitable habitat. We restricted pseudo-absences to a radius of 30 to 100km from presence records to account for environmental margins corresponding to reef slopes. This geographic distribution and the spatial resolution of the data restricted the number of pseudo-absences potentially available within three datasets, such that the recommended 10:1 pseudo-absence:presence ratio [41] could not be achieved. We then computed Pearson correlations between ensemble prediction metrics (mean, median and weighted mean) from each preliminary model to compare both predictions at a given pixel to assess differences and corresponding putative bias in predictions due to calibration area and associated differences in data collection and environmental data precision.

### Predictions

**Fig 2:**
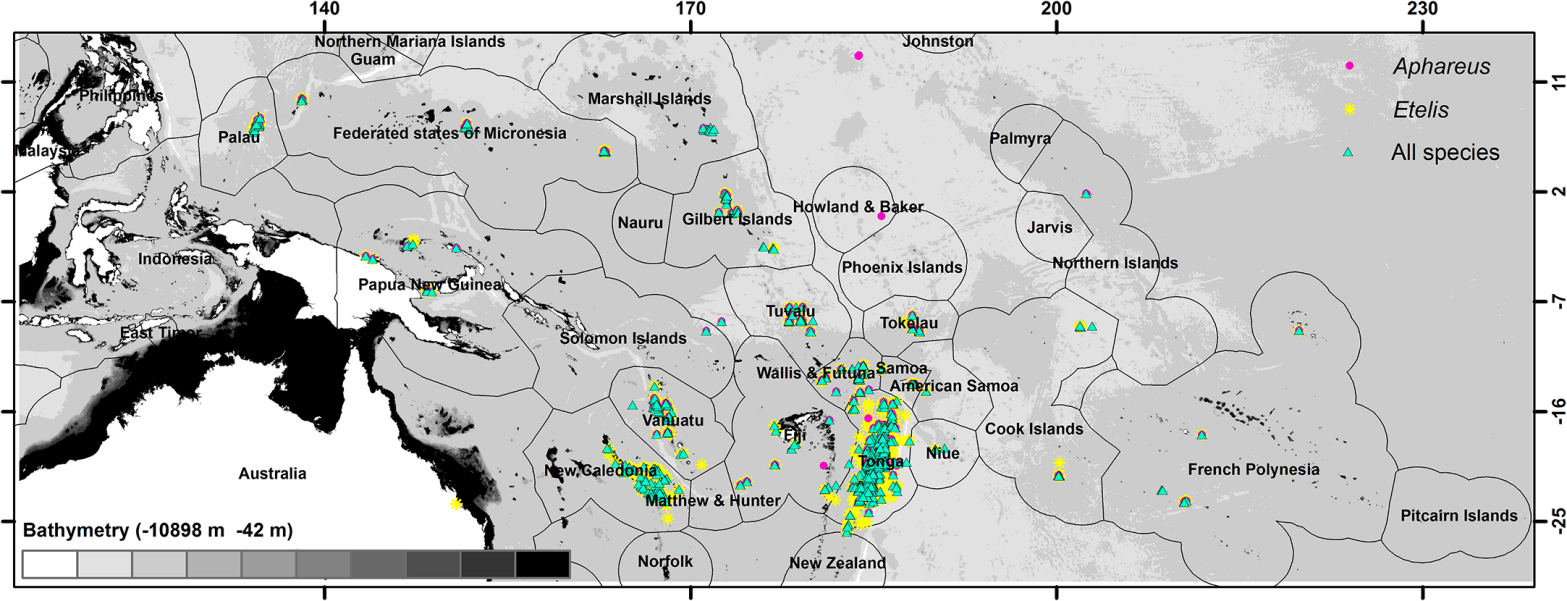
Location of presence data for deep-sea snapper across the Western Central Pacific Ocean. The locations for all *Pristipomoides* data are identical to those for *Aphareus* or *Etelis* and are indicated by all species.

To predict distributions of suitable habitat for each species group across the Western Central Pacific Ocean, we implemented regional distribution models at 0.25° resolution that included all available presence data. The complete presence dataset consisted of 2929 locations for *Etelis*, 1784 locations for *Pristipomoides* and 779 locations for *Aphareus* records, spread across 19 Pacific Island countries (Fig 2, Table 1). We generated new pseudo-absence records by randomly selecting across the studied area with a presence:pseudo-absence ratio of 1:10 [41]. To limit computation time, we randomly generated only two pseudo-absence datasets with five replications of each, implementing the same four algorithms as for the preliminary models.

**Table 1:** Number of locations included in the models for each country or territory where each species group was present.

Country or Territory	*Etelis*	*Pristipomoides*	*Aphareus*
American Samoa	1	1	0
Australia	1	0	0
Cook Islands	19	14	2
Federated states of Micronesia	18	28	22
Fiji	29	39	13
French Polynesia	6	9	6
Gilbert Islands (Kiribati)	25	36	20
Howland & Baker	0	0	1
Marshall Islands	0	12	7
New Caledonia	88	66	2
Niue	2	4	0
Northern Islands (Kiribati)	0	1	1
Palau	22	18	20
Papua New Guinea	9	10	6
Samoa	16	16	8
Solomon Islands	0	1	1
Tokelau	10	9	5
Tonga	2530	1355	592
Tuvalu	41	42	27
Vanuatu	48	57	19
Wallis & Futuna	64	66	27
Total	2929	1784	779

Regional ensemble predictions yielded probabilities of presence for each cell of the study area and for each species group. We selected a specific threshold to transform the probabilities to presence/absence and provide an easily transferable support for spatial management [42]. We applied the sensitivity/specificity equality threshold provided by MAXENT outputs to the predictions because it is not biased toward better prediction of presence or absence [43].

## Results

### Preliminary models

Independent model predictions varied slightly depending on the area of calibration (Tonga or New Caledonia) for *Etelis* and *Pristipomoides*. Model accuracy was generally higher for Tonga than for New Caledonia (Table 2), which could be due to the larger dataset available for the former. Precision (true skill statistics) was highest for the MAXENT algorithm for New Caledonia, while precision was highest for both the MAXENT and CTA algorithms for Tonga. The variation in precision between replicate model runs using the same pseudo-absence dataset was generally low (0.01–0.21). Correlations between ensemble predictions on the same region and calibrated on different data were high (r > 0.83, *p*< 0.0001) despite differences in presence data precision and distribution between New Caledonia and Tonga, and the different bathymetric datasets used (Table 3).

**Table 2:** True skill statistics for preliminary distribution models for *Etelis* Cuvier 1828, and *Pristipomoides* Valenciennes 1830 in New Caledonia and Tonga.

			PA_1_		PA_2_		PA_3_		Total	
Species group	Country	Algorithm	Mean	SD	Mean	SD	Mean	SD	Mean	SD
*Etelis*	New Caledonia	CTA	0.94	0.10	0.78	0.03	0.87	0.03	0.86	0.09
		MARS	0.91	0.05	0.81	0.08	0.77	0.29	0.83	0.16
		GLM	0.80	0.09	0.74	0.10	0.79	0.13	0.77	0.10
		MAXENT	0.95	0.05	0.86	0.05	0.94	0.03	0.92	0.06
	Tonga	CTA	0.99	0.01	0.98	0.01	0.98	0.00	0.98	0.01
		MARS	0.92	0.12	0.86	0.08	0.98	0.01	0.92	0.09
		GLM	0.98	0.01	0.96	0.01	0.96	0.01	0.97	0.01
		MAXENT	0.99	0.01	0.98	0.01	0.97	0.01	0.98	0.01
*Pristipomoides*	New Caledonia	CTA	0.90	0.06	0.87	0.05	0.94	0.06	0.90	0.06
		MARS	0.92	0.09	0.88	0.13	0.86	0.03	0.89	0.09
		GLM	0.84	0.10	0.91	0.07	0.84	0.05	0.86	0.08
		MAXENT	0.96	0.06	0.90	0.04	0.96	0.03	0.94	0.04
	Tonga	CTA	0.97	0.02	0.98	0.01	0.97	0.00	0.97	0.01
		MARS	0.98	0.01	0.99	0.01	0.98	0.01	0.98	0.01
		GLM	0.97	0.02	0.96	0.02	0.96	0.01	0.96	0.01
		MAXENT	0.97	0.01	0.98	0.01	0.98	0.01	0.98	0.01

PA_*i*_: pseudo-absence dataset*i*; CTA: classification tree analysis; MARS: multiple adaptive regression spline; GLM: generalized linear model; MAXENT: maximum of entropy; SD: standard deviation.

**Table 3:** Pearson correlation coefficients between ensemble model predictions calibrated on New Caledonia and Tonga data and then projected on either Tonga or New Caledonia for *Etelis* Cuvier 1828, and *Pristipomoides* Valenciennes 1830.

Species group	Projection	Mean	Median	Weighted mean
*Pristipomoides*	Tonga	0.89	0.84	0.89
	New Caledonia	0.96	0.95	0.96
*Etelis*	Tonga	0.83	0.83	0.83
	New Caledonia	0.94	0.94	0.94

All values had Type I errors < 0.001.

### Model Predictions across the Western Central Pacific Ocean

**Fig 3:**
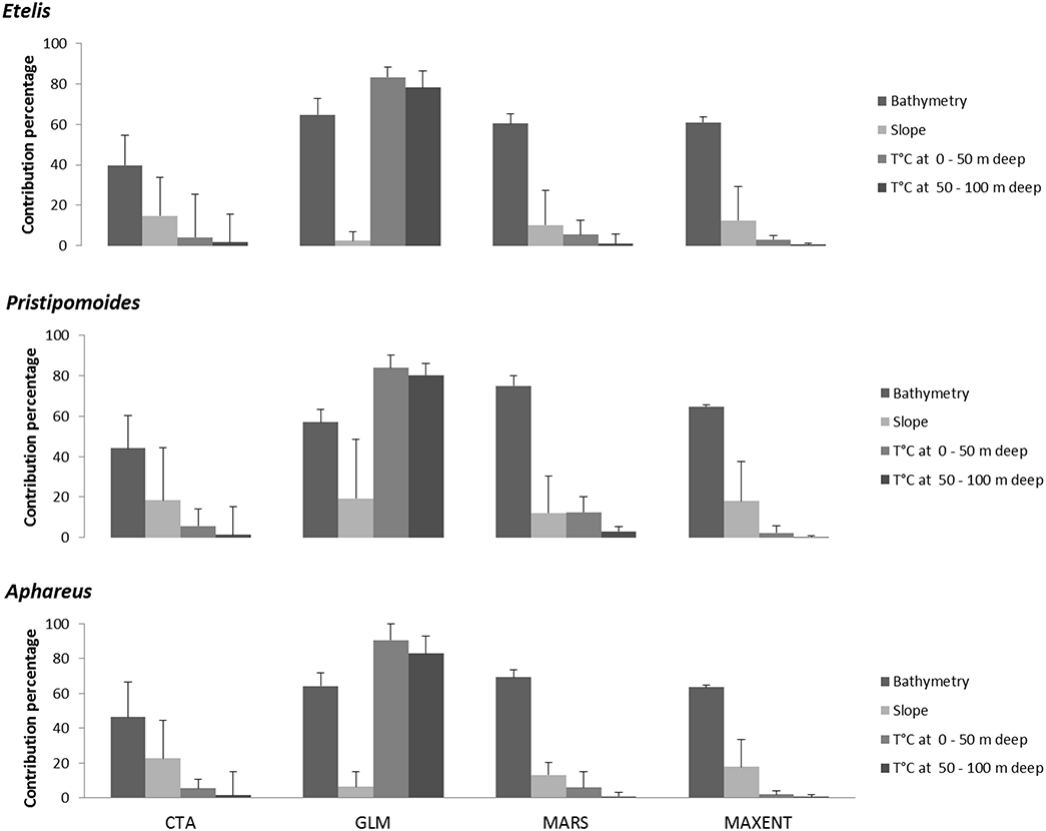
Relative contribution of oceanographic variables to model predictions calculated by resampling (*n* = 100) and averaged over pseudo-absence datasets (with corresponding standard deviation error bars) for each algorithm for *Etelis* Cuvier 1828, *Pristipomoides*Valenciennes 1830, and *Aphareus* Cuvier 1830. Month effect has been removed for temperature at depth (T°C). CTA: classification tree analysis; MARS: multiple adaptive regression spline; GLM: generalized linear model; MAXENT: maximum of entropy.

Depth was consistently the best predictor of presence for all species groups across all models except GLMs, but the relative contribution of other predictors was variable (Fig 3). Slope was a poor predictor of presence in all ensemble models for all species groups. Temperature at depth was a good predictor of presence for GLM, but was generally less important for other algorithms.

Regional model precision was high for the CTA, MAXENT and GLM algorithms (true skill statistic > 0.9), but lower and more variable for the MARS algorithm (true skill statistic range 0.33–0.79) (Table 4). Most of the MARS models were not included in the global ensemble modelling because we chose to use only independent models with a true skill statistic ≥0.7.

**Table 4:** True skill statistics for regional models for *Etelis* Cuvier 1828,*Pristipomoides* Valenciennes 1830, and *Aphareus* Cuvier 1830.

		PA_1_		PA_2_		Total	
Species group	Algorithm	Mean	SD	Mean	SD	Mean	SD
*Etelis*	CTA	0.93	0.00	0.93	0.00	0.93	0.00
	MARS	0.72	0.05	0.33	0.36	0.53	0.32
	GLM	0.94	0.00	0.93	0.00	0.94	0.00
	MAXENT	0.92	0.00	0.92	0.00	0.92	0.00
*Pristipomoides*	CTA	0.92	0.01	0.91	0.01	0.92	0.01
	MARS	0.66	0.20	0.79	0.12	0.73	0.17
	GLM	0.93	0.01	0.92	0.01	0.93	0.01
	MAXENT	0.92	0.01	0.91	0.01	0.91	0.01
*Aphareus*	CTA	0.91	0.01	0.90	0.01	0.91	0.01
	MARS	0.56	0.15	0.71	0.05	0.64	0.11
	GLM	0.93	0.01	0.93	0.01	0.93	0.01
	MAXENT	0.91	0.01	0.91	0.02	0.91	0.01

PA: pseudo absence dataset_*i*_; CTA: classification tree analysis; MARS: multiple adaptive regression spline; GLM: generalized linear model; MAXENT: maximum of entropy; SD: standard deviation.

**Fig 4:**
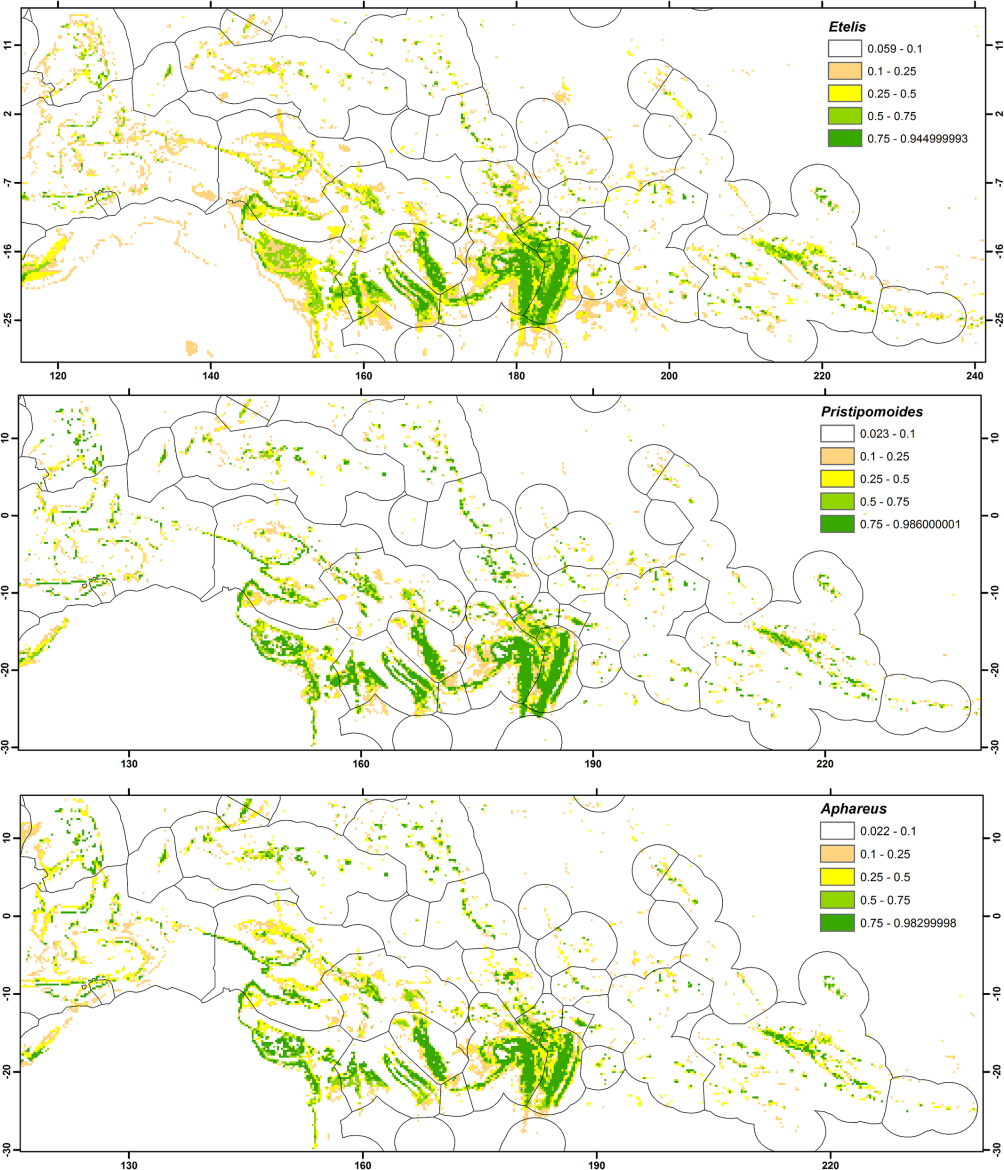
Projection of ensemble models in the Western Central Pacific region showing mean of probabilities of presence for *Etelis* Cuvier 1828, (upper panel), *Pristipomoides* Valenciennes 1830 (middle panel) and *Aphareus* Cuvier 1830 (lower panel), evaluated with true skill statistics and receiver operating characteristics (see Table 4).

The true skill statistic for the mean of probabilities was highest or equal highest across all four ensemble models for the 70% calibration dataset (Table 5). However, the differences were low (thousandth) and so we used the median of the probabilities (they are less sensitive to outliers) to map predictions of potential distribution for deep-sea snappers across the Western Central Pacific Ocean for *Etelis*, *Pristipomoides* and *Aphareus* (Fig 4). Although there were some differences in the distributions among species groups, most of the predicted suitable habitat overlapped. The equal test sensitivity and specificity thresholds produced by MAXENT were 0.084, 0.056 and 0.046 for *Etelis*, *Pristipomoides* and *Aphareus*, respectively. We used these thresholds to map the potential suitable habitat surfaces across the region assuming a binary presence/absence pattern.

**Table 5:** True skill statistics (TSS) for the four ensemble-model metrics implemented for *Etelis* Cuvier 1828, *Pristipomoides* Valenciennes 1830, and *Aphareus* Cuvier 1830.

Ensemble-models metrics	*Etelis*	*Pristipomoides*	*Aphareus*
	*TSS*	*TSS*	*TSS*
Mean of probabilities	0.948	0.938	0.946
Coefficient of variation of probabilities	0	0	0
Median of probabilities	0.946	0.934	0.941
Weighted mean of probabilities	0.948	0.938	0.945

There were strong regional patterns in the predicted distribution of suitable habitat for deep-sea snappers, with large areas of suitable habitat predicted in some Exclusive Economic Zones such as New Caledonia and Tonga, and more limited habitat predicted in others (Table 6). Suitable habitat area was calculated using the total area of 0.25° cells within which suitable habitat was predicted, so it provides an upper bound for actual habitat area. The highest proportion of suitable habitat was predicted in South Pacific countries, located between approximately 15 and 25°S. Over 70% of cells within Tonga’s Exclusive Economic Zone and at least 30% within the Exclusive Economic Zones of five other South Pacific countries or territories (Fiji, Wallis & Futuna, Vanuatu, New Caledonia, and Matthew & Hunter) were predicted to contain suitable habitat for all three deep-sea snapper species groups. In contrast, less than 5% of cells within the Exclusive Economic Zones of four countries or territories (Australia, Howland &Baker, Jarvis, and Nauru) were predicted to contain suitable habitat for all three species groups. The amount of predicted suitable habitat also varied among species groups, with the proportion of cells predicted to contain the highest suitable habitat for *Aphareus* and lowest for *Etelis* in almost all Exclusive Economic Zones (Table 6).

**Table 6:** Potential area (×10^3^km^2^) and proportion (prop) of suitable habitat of deep-sea snapper species within the Exclusive Economic Zones of 32 countries and territories based on higher-than-equal sensitivity-specificity thresholds (see text for details) from regional models at 0.25° spatial resolution.

Country or Territory	*Etelis*		*Pristipomoides*		*Aphareus*		Estimated unexploited biomass (*t*)
	area	prop	area	prop	area	prop	
American Samoa	18.5	0.04	23.1	0.06	30.8	0.07	—
Australia*	733.1	0.04	817	0.04	832.4	0.05	—
Cook Islands	85.5	0.04	139.4	0.07	244.9	0.12	413
East Timor	10.8	0.11	39.3	0.42	55.4	0.59	—
Federated states of Micronesia	90.1	0.03	301.9	0.10	410.4	0.14	1489
Fiji	714.6	0.50	828.6	0.58	914.1	0.64	4092
French Polynesia	429.7	0.08	571.4	0.11	662.3	0.12	3427
Gilbert Islands (Kiribati)^#^	44.7	0.04	91.6	0.09	97.8	0.09	731
Guam	13.9	0.06	47.7	0.21	95.5	0.42	22
Howland & Baker	0.8	0.00	12.3	0.29	21.6	0.05	—
Indonesia*	224.1	0.03	834.7	0.11	1271.4	0.16	—
Jarvis	0	0.00	0	0.00	9.2	0.03	—
Marshall Islands*	42.4	0.02	172.5	0.08	274.1	0.13	1108
Matthew & Hunter	90.1	0.38	84.7	0.35	67	0.28	—
Nauru	1.5	0.50	1.5	0.50	3.1	0.01	3
New Caledonia	517.5	0.41	504.4	0.40	471.3	0.37	1089
Niue	26.2	0.08	24.6	0.07	50.8	0.15	70
Northern Islands (Kiribati)^#^	33.1	0.02	91.6	0.06	135.5	0.08	731
Northern Mariana Islands*	9.2	0.01	23.9	0.03	43.1	0.05	236
Palau	10	0.02	32.3	0.05	50.1	0.08	162
Palmyra	4.6	0.02	35.4	0.12	44.7	0.15	—
Papua New Guinea	363.5	0.13	736.2	0.25	944.9	0.33	4881
Philippines	110.1	0.05	194.1	0.09	276.5	0.12	—
Phoenix Islands (Kiribati)^#^	23.1	0.03	57.8	0.08	64.7	0.09	731
Pitcairn Islands	51.6	0.05	53.9	0.05	46.2	0.05	11
Samoa	22.3	0.16	37	0.27	41.6	0.30	190
Solomon Islands	205.6	0.12	463.6	0.28	606	0.36	1711
Tokelau	15.4	0.04	39.3	0.11	64.7	0.18	99
Tonga	528.3	0.72	551.4	0.75	557.5	0.76	1125
Tuvalu	97	0.13	177.9	0.23	249.5	0.33	224
Vanuatu	250.3	0.35	301.1	0.42	345	0.48	980
Wallis & Futuna	127.1	0.48	147.9	0.56	153.2	0.58	102

*partially covered by the present model.

^#^Biomass estimate derived from all three EEZ areas

Note that potential area was calculated using the total area of 0.25° cells within which suitable habitat was identified and, therefore, provides an upper bound for true habitat area. Estimates of unexploited biomass were available for the Exclusive Economic Zones of 23 countries and territories (Dalzell & Preston, 1992).

## Discussion

We have provided the first model predictions of deep-sea snapper habitat suitability in the Western Central Pacific Ocean. Our results demonstrate that despite data paucity, which is a common feature for most offshore fisheries and is a characteristic of the deep-sea fisheries in this region, the relationship between deep-sea snapper catch data and readily available environmental attributes was sufficiently strong to predict their presence across a large area encompassing the Exclusive Economic Zones of 32 countries and territories. The strength of this relationship was also sufficiently robust at relatively low precision (0.25°) in environmental variables. Consequently, the data paucity associated with the fisheries and environmental variables should not prevent the application of distribution modelling in marine spatial planning for this species assemblage.

**Fig 5:**
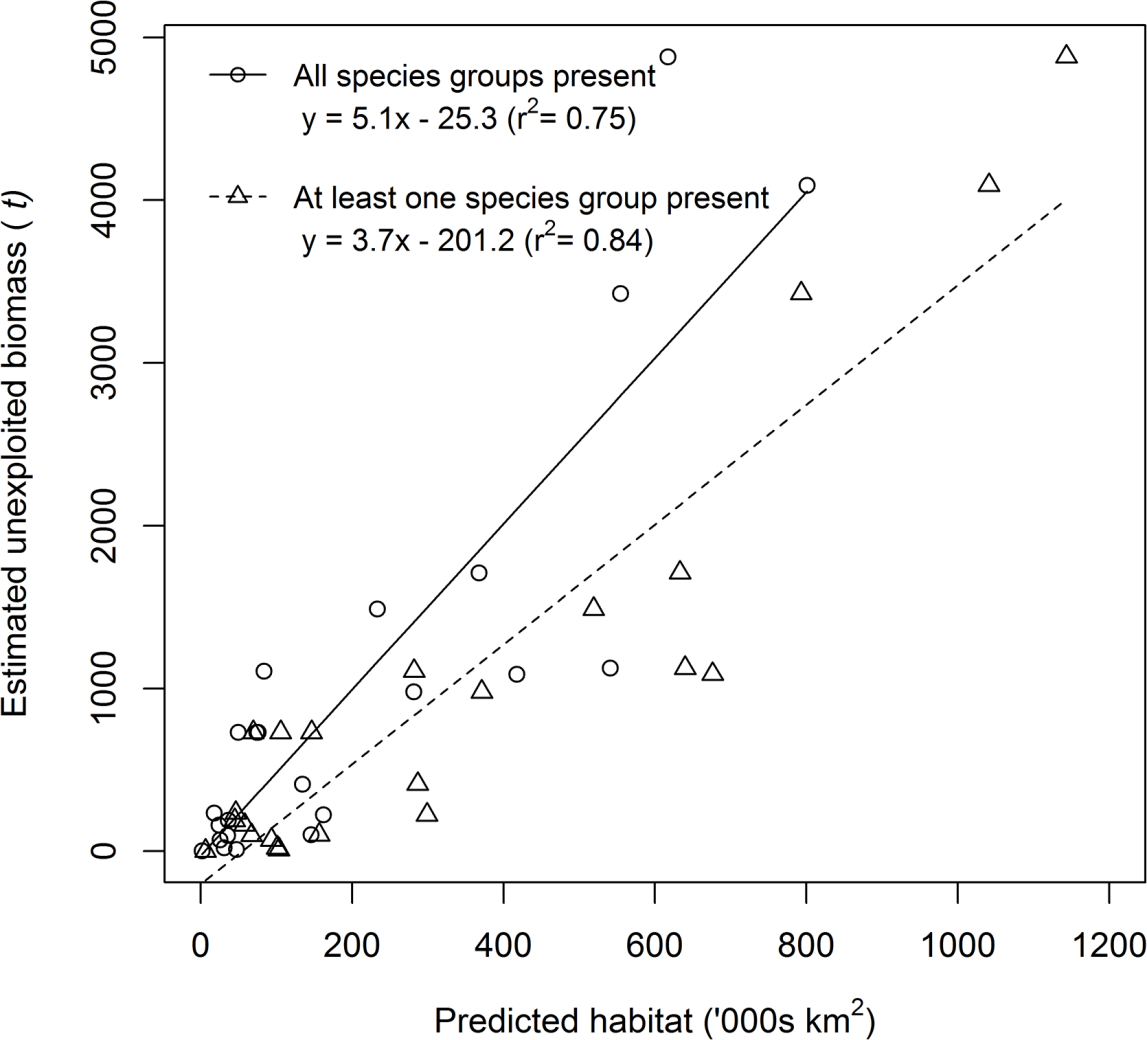
Relationship between estimated unexploited biomass (source: Dalzell & Preston, 1992) and predicted suitable habitat of deep-sea snappers. Data are shown for predicted habitat when all three species groups (*Etelis*, *Pristipomoides*, *Aphareus*) and at least one species group were predicted to be present.

Knowledge of the distribution of exploited species is essential for policy makers to make informed decisions about developing new fisheries or managing existing ones. The large variation in area of predicted deep-sea snapper habitat among countries and territories has important implications for the development and management of deep-sea fisheries in this region. For example, the largest area of predicted habitat was in the South Pacific, mostly within the Exclusive Economic Zones of those countries that have established deep-sea snapper fisheries, either currently or historically. Our results suggest that there might be limited scope for development of new deep-sea snapper fisheries in some countries and territories where the area of predicted habitat suitability was low. However, predicted habitat suitability from presence data does not consider abundance, and so it will be necessary to obtain reliable information on the local abundance of species to estimate potential yields. Rudimentary assessments of deep-sea snappers in the Pacific region provide preliminary estimates of unexploited biomass for 23 Pacific Island countries based on data from depletion experiments and estimates of the length of the 200 m isobaths within each country [43]. Indeed, there was evidence for a positive relationship between estimated unexploited biomass and predicted habitat area (Fig 5; Table 6), supporting our assertion that opportunities are likely to be limited for development of deep-sea snapper fisheries in countries and territories where predicted suitable habitat area is low.

We predicted suitable habitat for the most commonly harvested deep-sea snappers; however, the extent of this habitat varied among species groups, highlighting potential differences in habitat selection among deep-sea snapper groups. Most species are distributed over a wide depth range from approximately 50–400 m [28]. However, underwater observations (restricted to a maximum depth of 300 m) from the main Hawaiian Islands found that *Etelis* are more abundant at210–300 m and *Pristipomoides* are more abundant at 90–270 m [31, 32]. There are no observational data available for *Aphareus*, but individuals have been captured at depths between 50 and 350 m (A. J. Williams, Secretariat of the Pacific Community, New Caledonia, unpublished data). A wider depth selection for *Pristipomoides*, and potentially *Aphareus*, likely explains the greater area of predicted habitat for these species in the Western Central Pacific Ocean compared to *Etelis*.

Deep-sea snapper habitat suitability was most strongly correlated with depth, which is consistent with underwater observations from Hawaii [31, 32]. We found that bathymetric slope was a poor predictor of deep-sea snapper habitat suitability, a finding inconsistent with observations in Hawaii where slope had a measurable effect (albeit less than the effect of depth)on abundance of some deep-sea snapper species [31]. This disparity, which might not be surprising as we did not model abundance but only presence, could arise because of divergent spatial scales between studies and the steepness of some deep-sea snapper habitats. The bathymetry data we used was available at a resolution of 0.016° (> 1000 m^2^), whereas Misa *et al*. [24] classified habitat at a scale of 200 m^2^. The coarser spatial scale data we used might not have captured the finer spatial variation in bathymetry and the heterogeneity in deep-sea snapper habitat. For more precise predictions of deep-sea snapper habitat, it will be necessary to obtain bathymetric data at a finer spatial scale, the cost of which is likely prohibitive for most Pacific Island countries and territories. The predictions we present here, however, provide a useful baseline from which future bathymetric mapping can be prioritized.

An underlying assumption of species distribution models is that the species is at equilibrium with its environment and that relevant environmental gradients have been adequately sampled [1]. Deep-sea snappers are strongly associated with specific benthic habitats[31]and make only minimal (<10 km)horizontal movements [44], which lends support for this assumption. In scenarios where pseudo-absences are used because no true absence data are available, it is likely that the data describe only a subset of the ecological processes shaping the species’ distribution, and subsequent predictions are affected by the location of the pseudo-absences [45, 46]. We were able to test this potential bias partially through a model validation process, which revealed that neither the differences in quality of the fisheries or oceanographic layer data (mainly bathymetry) substantially affected model predictions. That is, the model developed using pseudo-absence data from one region captured a similar component of the species distributions in another. The extent to which the pseudo-absence data are robust in other regions of the Western Central Pacific Ocean unknown because the model validation was limited to Tonga and New Caledonia. However, we applied a ratio of 1:10 for presence:pseudo-absence records [47] to limit potential errors due to a lack of representative pseudo-absence data across the environmental background.

Assessing model uncertainty and evaluating model limitations are necessary to understand cumulative errors, particularly if the models are to be used in management. The ensemble approach we applied provides a method to account for the uncertainty in predictions and data, and appears particularly adapted to the spatially clumped data characteristic of most fisheries. Indeed, an ensemble approach can overcome single-method statistical limits and combine multiple-method performances. To be able to use such predictions for conservation planning, particular attention to commission (false positives) and omission (false negatives) errors is essential [48]. Conservation targets for habitat types strongly depend on sample size and sampling effort [49]. Conservation planning in remote oceanic regions is also biased towards surveyed and exploited sites given incomplete data coverage. Species distribution modelling might be a useful method for better estimating the extent of deep-sea snapper habitats, providing direction for future protected area establishment and a means of assessing the effectiveness of protected area networks [50].

## Conclusion

Our results represent a useful baseline for designing monitoring programs that balance the often-divergent aims of resource exploitation and conservation planning. Integrating monitoring programs at the scale of the entire Western Central Pacific Ocean through a multinational collaboration has clear benefits [24]. Collaboration in management among countries could reduce the costs and increase conservation efficiency in the management of deep-sea snapper resources.

## Supporting Information

S1 Dataset Positions (latitude and longitude in decimal degrees) where deep-sea snapper species from three genera were captured during research surveys cross the territorial waters of 19 countries of the Western Central Pacific Ocean or by commercial fisheries from Tonga and New Caledonia. (XLSX)
